# Meningococcal ACWY conjugate vaccine immunogenicity in adolescents with primary or secondary immune deficiencies, a prospective observational cohort study

**DOI:** 10.1186/s12969-023-00846-3

**Published:** 2023-07-20

**Authors:** Milou Ohm, Joeri W van Straalen, Gerrie de Joode-Smink, Joris van Montfrans, Marije Bartels, Joanne G van Wildenbeest, Caroline A Lindemans, Roos AW Wennink, Joke H de Boer, Elisabeth AM Sanders, Frans M Verduyn-Lunel, Guy AM Berbers, Nico M Wulffraat, Marc H.A. Jansen

**Affiliations:** 1 Centre for Infectious Disease Control, National Institute for Public Health and the Environment (RIVM), Bilthoven, the Netherlands; 2 Department of Pediatric Immunology and Rheumatology, Wilhelmina Children’s Hospital, University Medical Centre Utrecht, Utrecht, the Netherlands; 3 Department of Pediatric Hematology, Wilhelmina Children’s Hospital, University Medical Centre Utrecht, Utrecht, the Netherlands; 4 Department of Pediatric Infectious Diseases, Wilhelmina Children’s Hospital, University Medical Centre Utrecht, Utrecht, the Netherlands; 5 Princess Máxima Center for Pediatric Oncology, Utrecht, The Netherlands; 6 Department of Ophthalmology, University Medical Centre Utrecht, Utrecht, the Netherlands; 7 Department of Medical Microbiology, University Medical Centre Utrecht, Utrecht, The Netherlands

**Keywords:** MenACWY conjugate vaccination, Antibody responses, Immunodeficiency, Autoimmune disease, Inflammatory disease, Immunocompromised, Adolescents

## Abstract

**Background:**

Immunization with meningococcal ACWY conjugate vaccine induces protective antibodies against invasive meningococcal disease (IMD) caused by serogroups A, C, W and Y. We studied MenACWY-TT vaccine immunogenicity in adolescents with a heterogenous group of primary and secondary immune deficiency including patients with systemic lupus erythematosus, mixed connective tissue disease, vasculitis, uveitis, 22Q11 syndrome, sickle cell disease, and patients who underwent stem cell transplantation for bone marrow failure.

**Findings:**

We enrolled 69 individuals aged 14–18 years diagnosed with a primary or secondary immune deficiency in a prospective observational cohort study. All patients received a single dose of MenACWY-TT vaccine during the catch-up campaign 2018-19 because of the IMD-W outbreak in the Netherlands. Capsular polysaccharide-specific (PS) IgG concentrations against MenACWY were measured before and 3–6, 12, and 24 months after vaccination. Overall, geometric mean concentrations (GMCs) of MenACWY-PS-specific IgG were lower in patients compared to data from healthy, aged-matched controls (n = 75) reaching significance at 12 months postvaccination for serogroup A and W (adjusted GMC ratios 0.26 [95% CI: 0.15–0.47] and 0.22 [95% CI: 0.10–0.49], respectively). No serious adverse events were reported by study participants.

**Conclusions:**

The MenACWY conjugate vaccine was less immunogenic in adolescent patients with primary or secondary immunodeficiency compared to healthy controls, urging the need for further surveillance of these patients and supporting considerations for booster MenACWY conjugate vaccinations in these patient groups.

**Supplementary Information:**

The online version contains supplementary material available at 10.1186/s12969-023-00846-3.

## Introduction

Individuals with primary or secondary immunodeficiencies are more susceptible for a severe course of infections, because of their underlying disease and the use of immunomodulating medication that compromises immune defense against infection [1]. The spectrum of primary immunodeficiencies is large and includes humoral immune deficiency (such as common variable immunodeficiencies (CVID), cellular and combined immune deficiencies (CID), complement disorders, and (functional) asplenia. Secondary immunodeficiency is often due to immunosuppressive treatment and therefore can occur in a wide spectrum of inflammatory diseases, such as juvenile idiopathic arthritis (JIA) and inflammatory bowel disease (IBD), but may also be caused by more rare diagnoses including mixed connective tissue disorder (MCTD), systemic lupus erythematosus (SLE), as well as several forms of vasculitis. Prevention of infections is crucial to reduce the number of (intensive care) admissions and mortality of invasive bacterial infections such as (vaccine-preventable) meningococcal and pneumococcal disease [2–4]. Data on immunogenicity of polysaccharide-conjugate vaccines in medical high-risk groups such as immunocompromised patients remain relatively scarce [5, 6].

In 2018, a national outbreak of serogroup W invasive meningococcal disease (IMD-W) in the Netherlands urged the implementation of a meningococcal serogroup A, C, W and Y (MenACWY) conjugate vaccination at 14 years of age, next to a catch-up campaign for individuals 14–18 years [7]. We conducted a prospective observational study on antibody levels before and after MenACWY vaccination in a cohort of individuals diagnosed with primary or secondary immunodeficiency aged 14–18 years, with a follow-up of two years.

## Methods

Adolescents with immune disorders from the Wilhelmina Children’s Hospital of the University Medical Centre Utrecht were recruited for this prospective observational cohort study. For the current study, we enrolled patients with primary and secondary immunodeficiencies, excluding juvenile idiopathic arthritis (JIA) or inflammatory bowel disease (IBD) as these data were described separately (manuscript submitted). As part of the nationwide catch-up campaign in 2018 for individuals aged 14–18 years in the Netherlands, participants received a single dose of MenACWY-TT (Nimenrix®) from their local public health centers [7]. All patients had received primary MenC-TT conjugate vaccine at 14 months of age according to the national immunization programme (NIP). Clinical data and blood collection were combined with routine outpatient follow-up visits. Blood samples were collected before vaccination and at 3–6 months, 12 months (+/- 3 months) and 24 months (+/- 3 months) after vaccination. We measured MenACWY polysaccharide (PS)-specific serum IgG concentrations with a fluorescent bead-based multiplex immunoassay (MIA), as previously described [8]. Data were compared with published data from healthy, aged-matched controls (HCs) who participated in a randomized controlled trial, in which antibody concentrations were determined by the same laboratory according to the same procedures [9, 10]. For safety measurements, we evaluated self-reported (serious) adverse events postvaccination. Written informed consent was obtained from participants and caregivers (for patients < 16 years).

Patient characteristics at baseline (sex, age, disease category and medication use) were presented as frequency with percentage for categorical variables and median with interquartile range (IQR) for numerical variables. For all analyses, MenACWY-PS specific IgG concentrations were log-transformed prior to analysis and presented as geometric mean concentrations (GMCs) with 95% confidence intervals (CI). In order to adjust for baseline differences, GMCs were compared between study participants and HCs at 12 months postvaccination using a multivariable linear regression analysis. The regression coefficient was exponentiated to obtain (adjusted) GMC ratios with 95% CIs for study participants versus HCs. GMCs in the total patient cohort were compared between study visits for each serogroup using pairwise t-tests with Bonferroni correction. GMCs were compared between boys and girls within the study participant cohort at all study visits using the t-test. GMCs within patients were compared between the different diseases, as well as between three large disease subgroups (primary immunodeficiency, secondary immunodeficiency, hematological condition) at all timepoints using ANOVA test. For all analyses, a p-value of < 0.05 was considered statistically significant. All analyses were performed using R version 4.0.

## Results

We included 82 patients in the current study between October 2018 and March 2020. For 13 patients no serological data were collected and these patients were excluded from further analyses. The median age of participants was 15.3 years, and approximately half of the remaining 69 participants were female (49%) (Table 1).

**Table 1:** Patient characteristics at baseline

Characteristics	Total cohort (n = 69)	Healthy controls (n = 75)
Female, n (%)	34 (49.3%)	36 (48.0%)
Age in years, median (IQR)	15.3 (13.7–17.0)	15·2 (14·9–15·5)
Disease, n (%)		N/A
*Immune deficiencies* ^*1*^	33 (47.8%)	
*Autoimmune and auto-inflammatory diseases* ^*2*^	20 (29.0%)	
*Uveitis*	12 (17.4%)	
*Sickle cell disease*	4 (5.8%)	
Medication use, n (%)		
*NSAIDs*	5 (7.2%)	
Immunosuppressive drugs*	23 (33.3%)	
*Systemic corticosteroids*	1 (1.4%)	
*Synthetic DMARDs*	19 (27.5%)	
*Methotrexate*	6 (8.7%)	
*Azathioprine*	1 (1.4%)	
*Mycophenolate mofetil*	11 (15.9%)	
*Other*	1 (1.4%)	
*Biologic DMARDs*	10 (14.5%)	
*Anti-TNF*	7 (10.1%)	
*Anti-IL6*	2 (2.9%)	
*Anti-IL1*	1 (1.4%)	

Abbreviations: DMARDS, disease-modifying anti-rheumatic drugs; IQR, interquartile range; N/A, not applicable. *including systemic corticosteroids, synthetic DMARDS, biologic DMARDS.

^1^11 common variable immunodeficiency; 8 22Q11 syndrome; 3 chronic neutropenia; 3 specific polysaccharide antibody deficiency; 2 complement 2 deficiency; 1 bone marrow failure/allogeneic stem cell transplantation; 1 hyper IgE syndrome; 1 warts, hypogammaglobulinemia, infections and myelokathexis syndrome; 1 ataxia telangiectasia; 1 dysimmunoglobulinemia; 1 IgG subclass deficiency.

^2^ 6 systemic lupus erythematosus; 3 mixed connective tissue disease; 3 juvenile dermatomyositis; 2 systemic sclerosis; 1 recurrent idiopathic pericarditis; 1 chronic recurrent multifocal osteomyelitis; 1 localized scleroderma; 1 eosinophilic granulomatosis with polyangiitis; 1 alopecia areata with dysimmunoglobulinemia; 1 adenosine deaminase 2 deficiency.

**Fig. 1:**
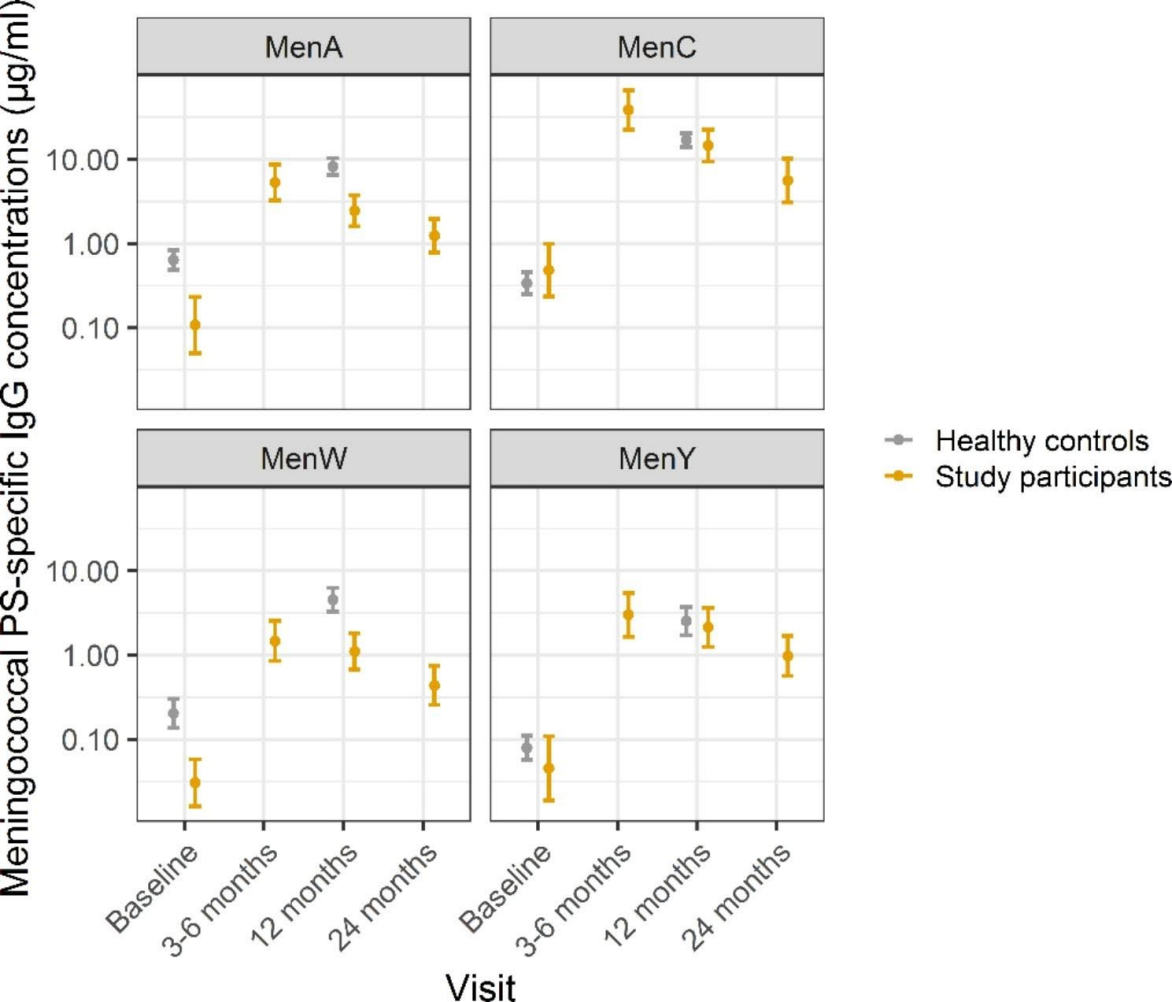
Geometric mean concentrations of meningococcal serogroup A, C, W and Y (MenACWY) polysaccharide (PS)-specific serum antibody concentrations of study participants and healthy controls during follow-up. Dots indicate geometric mean concentrations with 95% confidence interval

**Fig. 2:**
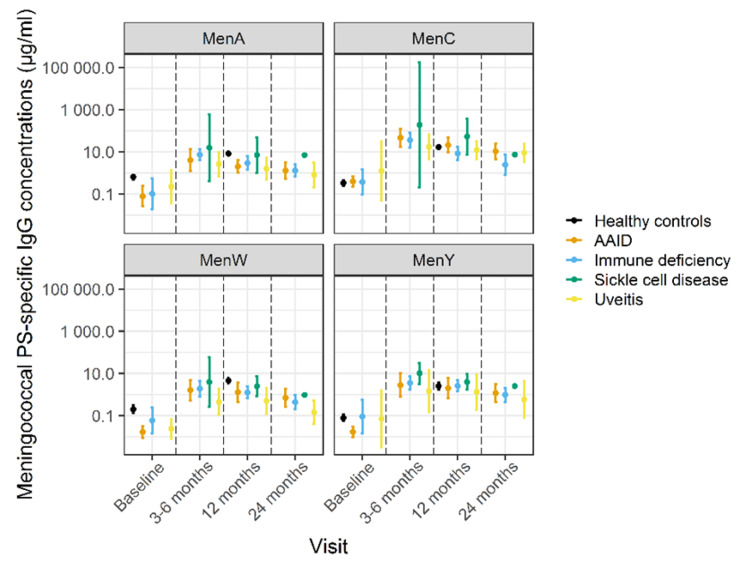
Meningococcal serogroup A, C, W and Y polysaccharide (PS)-specific serum IgG concentrations in patients and healthy controls during follow-up. Dots indicate geometric mean concentrations with 95% confidence intervals. Abbreviations: AAID, autoimmune and auto-inflammatory diseases

At baseline, GMCs of ≤ 0.5 µg/mL were observed for all serogroups (Table 2) with GMCs in the patients significantly lower compared with HCs for serogroup A and W, but not for serogroup C and Y (Fig. 1). GMCs increased for all serogroups at three months postvaccination, the highest increase observed for serogroup C, which concerns a booster vaccination. Compared with HCs at 12 months postvaccination, GMCs were lower (p < 0.01) for serogroup A and W (2.5 and 1.1 µg/mL versus 8.2 and 4.5 µg/mL respectively) but not significantly different for C and Y (14.6 and 2.1 µg/mL versus 16.9 and 2.5 µg/mL respectively) (Fig. 1; Table 2). Differences for serogroup A and W continued to exist after adjusting for baseline IgG concentrations with a linear regression model (adjusted GMC ratios 0.26 [0.15–0.47] and 0.22 [0.10–0.49], respectively) (Supplementary Table 1). GMCs showed a decreasing trend between 3 and 6 to 12 months and between 12 and 24 months postvaccination for all serogroups (Fig. 2), albeit not significant for A, W and Y (Supplementary Table 2). We did not find differences in GMCs postvaccination between female and male subjects within the patient cohort (Supplementary Table 3).

Overall, differences between the various disease groups were limited, though patient subgroups were small (Fig. 2 and Supplementary Table 4). GMCs showed a higher trend in patients with sickle cell disease compared to patients with primary or secondary immunodeficiency at 12 months postvaccination (Fig. 2, p < 0.05 for serogroup A and C, not significant for serogroup W and Y), but the sample size for each group was very small.

**Table 2:** Geometric mean concentrations and 95% confidence intervals of MenACWY PS-specific IgG concentrations (µg/ml) during follow-up

Months	Serogroup	Study participants	Healthy controls	*P*
0		n = 26	n = 75	
	MenA	0.1; 0.1–0.2^1^	0.6; 0.5–0.8^1^	< 0.01*
	MenC	0.5; 0.2–1.0	0.3; 0.3–0.5	0.36
	MenW	0.0; 0.0–0.1	0.2; 0.1–0.3	< 0.01*
	MenY	0.0; 0.0–0.1	0.1; 0.1–0.1	0.23
3–6		n = 45	n = 0	
	MenA	5.3; 3.3–8.7	-	-
	MenC	38.5; 22.5–65.8	-	-
	MenW	1.5; 0.9–2.5	-	-
	MenY	3.0; 1.7–5.5	-	-
12		n = 47	n = 75	
	MenA	2.5; 1.6–3.7	8.2; 6.5–10.4	< 0.01*
	MenC	14.6; 9.4–22.5^1^	16.9; 14.0–20.4	0.53
	MenW	1.1; 0.7–1.8	4.5; 3.3–6.3	< 0.01*
	MenY	2.1; 1.3–3.7	2.5; 1.7–3.8	0.60
24		n = 39	n = 0	
	MenA	1.2; 0.8–2.0	-	-
	MenC	5.6; 3.1–10.2	-	-
	MenW	0.4; 0.3–0.7	-	-
	MenY	1.0; 0.6–1.7	-	-

*P < 0.05

^1^one missing observation

Two patients reported an event of special interest after vaccination: one patient reported a transient headache and another patient (who received a concomitant influenza vaccination) fainted after the vaccination with spontaneous recovery; this patient also reported a transiently enlarged lymph node. No serious adverse events were reported.

## Discussion

Our study found a lower IgG antibody response to a single primary MenACWY-TT conjugate vaccine in immunocompromised adolescents compared to HCs against serogroup A and W, but not for the booster vaccination to serogroup C. Serious adverse events following the MenACWY vaccination were not reported in the current study population.

To our knowledge, no studies have reported results on the meningococcal conjugate vaccine response in autoimmune inflammatory rheumatic disease (AIIRD), other than two studies on MenC conjugate vaccination in JIA patients [5, 11, 12]. Our findings are consistent with a study in JIA and IBD patients, showing a reduced functionality of antibodies and a lower proportion of protecting antibodies against serogroup W following MenACWY vaccination; differences in that study were especially pronounced in anti-TNF users [13]. Data in literature concerning immunocompromised individuals are scarce, including groups at high risk for IMD such as individuals with complement deficiencies or use of eculizumab [14–16]. In the Netherlands, one booster is advised 3–5 years after the primary single MenACWY and MenB vaccination for asplenic individuals aged 1–24 years [17]. For complement deficiencies, repeat boosters every 5 years are advised. However, for other primary immunodeficiencies and for both pediatric and adult AIIRD patients, there are currently no recommendations on meningococcal vaccines available [18, 19]. Generally, the European Alliance of Associations for Rheumatology recommends to follow the NIP for pediatric AIIRD patients [6], but our studies suggest extra vaccination may be required for some individuals with immunodeficiency. Recommendations based on studies in the larger patient groups (JIA, IBD) are often extended to all AIIRDs because of a lack of data rather than similar immune pathology. This highlights the importance of further research into all (rare) diseases to improve protection against vaccine-preventable diseases in immunocompromised individuals and should include the immediate vaccine response as well as waning of antibodies over time as differences in kinetics have been described compared with HCs [11]. Importantly, albeit a small sample size, no severe adverse events were reported by the patients in our cohort in the first three months postvaccination. This confirms the assumption that meningococcal conjugate vaccines are safe in patients with immunodeficiencies.

Given the design of the study, we included a heterogenous patient cohort with a variety of (rare) immune disorders with low numbers for each separate disease and medication group, which hampered assessing differences between medication groups. Because sampling was dependent on regular hospital visits, a number of serum samples were lacking at different timepoints. We only assessed serogroup-specific IgG concentrations, while addition of a functional assay [20] would have been of value because functional circulating antibodies are crucial in the prevention of IMD. A strength of the study was that we compared with data from age-matched HCs from the same laboratory and using the same procedures.

Based on our observations, it seems sensible to consider a second MenACWY-TT dose for these immunocompromised groups. This however warrants further study and follow-up of these vulnerable patient groups.

## Electronic supplementary material

Below is the link to the electronic supplementary material.

Supplementary Material 1

Supplementary Material 2

## Data Availability

All relevant data are reported in the article. Additional details can be provided by the corresponding author upon reasonable request.

## Abbreviations

AAID: autoimmune and auto-inflammatory disease
CID: combined immune deficiencies
CVID: common variable immunodeficiencies
DMARDS: disease-modifying anti-rheumatic drugs
GMC: geometric mean concentration
HC: healthy controls
IBD: inflammatory bowel disease
IMD: invasive meningococcal disease
IQR: interquartile range
JIA: juvenile idiopathic arthritis
MenACWY: meningococcal serogroup ACWY
MCTD: mixed connective tissue disorder
MIA: multiplex immunoassay
NIP: national immunization programme
NSAID: non-steroidal anti-inflammatory drugs
PS: polysaccharide
SLE: systemic lupus erythematosus
anti-TNF: anti-tumor necrosis factor
