# Supplementary material for: Meningococcal ACWY conjugate vaccine immunogenicity in adolescents with primary or secondary immune deficiencies, a prospective observational cohort study
Source: Pediatr Rheumatol Online J. 2023 Jul 20;21:73. doi: 10.1186/s12969-023-00846-3 (PMC10360259; doi:10.1186/s12969-023-00846-3)
Supplement: Supplementary file 1 — Supplementary Material 1 [file 12969_2023_846_MOESM1_ESM.docx]

**SUPPLEMENTARY MATERIAL**

**Supplementary Table 1**. **Linear regression analyses for the geometric mean concentration (GMC) ratio of** **meningococcal serogroup ACWY polysaccharide-specific serum IgG concentrations at 12 months postvaccination for study participants versus healthy controls.**

| Serogroup | Analysis | GMC ratio for study participants  vs. healthy controls (95% CI) |
| --- | --- | --- |
| MenA | Crude | 0.30 (0.19 – 0.46)* |
|  | Adjusted^1^ | 0.26 (0.15 – 0.47)* |
| MenC | Crude | 0.86 (0.57 – 1.29) |
|  | Adjusted^1^ | 0.97 (0.58 – 1.61) |
| MenW | Crude | 0.24 (0.14 – 0.42)* |
|  | Adjusted^1^ | 0.22 (0.10 – 0.49)* |
| MenY | Crude | 0.84 (0.44 – 1.59) |
|  | Adjusted^1^ | 0.64 (0.23 – 1.74) |
| *statistically significant effect  ^1^adjusted for baseline IgG concentration | | |

**Supplementary Table 2. *P*-values for pairwise comparisons of meningococcal serogroup ACWY polysaccharide-specific serum IgG concentrations at different study visits for all study participants.**

| Serogroup |  | Time-point | Baseline | 3-6 months | 12 months |
| --- | --- | --- | --- | --- | --- |
| MenA |  | 3-6 months | <0.01* | - | - |
|  |  | 12 months | <0.01* | 0.11 | - |
|  |  | 24 months | <0.01* | <0.01* | 0.28 |
| MenC |  | 3-6 months | <0.01* | - | - |
|  |  | 12 months | <0.01* | 0.05* | - |
|  |  | 24 months | <0.01* | <0.01* | 0.07 |
| MenW |  | 3-6 months | <0.01* | - | - |
|  |  | 12 months | <0.01* | 1.00 | - |
|  |  | 24 months | <0.01* | <0.01* | 0.08 |
| MenY |  | 3-6 months | <0.01* | - | - |
|  |  | 12 months | <0.01* | 1.00 | - |
|  |  | 24 months | <0.01* | 0.05* | 0.35 |

**P* < 0.05
*P*-values were adjusted with Bonferroni correction

**Supplementary Table 3. Geometric mean concentrations and 95% confidence intervals of meningococcal serogroup ACWY polysaccharide-specific serum IgG concentrations (µg/ml) for study participants during follow-up stratified by sex.**

| Months | Serogroup | Girls | Boys | *P* |
| --- | --- | --- | --- | --- |
| 0 |  | n = 15 | n = 11 |  |
|  | MenA | 0.1; 0.0 – 0.1 | 0.2; 0.1 – 1.1^1^ | 0.12 |
|  | MenC | 0.3; 0.2 – 0.7 | 0.8; 0.2 – 3.6 | 0.32 |
|  | MenW | 0.0; 0.0 – 0.0 | 0.1; 0.0 – 0.3 | 0.02* |
|  | MenY | 0.0; 0.0 – 0.0 | 0.2; 0.0 – 1.2 | <0.01* |
| 3-6 |  | n = 25 | n = 20 |  |
|  | MenA | 5.6; 2.8 – 11.2 | 5.0;2.3 – 10.7 | 0.82 |
|  | MenC | 47.9; 20.9 – 110.1 | 29.3; 14.8 – 57.7 | 0.35 |
|  | MenW | 1.6; 0.7 – 4.1 | 1.3; 0.7 – 2.3 | 0.66 |
|  | MenY | 3.0; 1.2 – 7.5 | 3.0; 1.3 – 6.8 | 0.98 |
| 12 |  | n = 24 | n = 23 |  |
|  | MenA | 2.2; 1.2 – 4.2 | 2.8; 1.5 – 5.0 | 0.60 |
|  | MenC | 19.3; 10.2 – 36.5 | 10.7; 5.8 – 19.9^1^ | 0.17 |
|  | MenW | 1.2; 0.6 – 2.6 | 1.0; 0.5 – 2.0 | 0.63 |
|  | MenY | 2.3; 1.1 – 4.7 | 2.0; 0.8 – 4.7 | 0.79 |
| 24 |  | n = 18 | n = 21 |  |
|  | MenA | 1.2; 0.5 – 2.8 | 1.3; 0.7 – 2.2 | 0.96 |
|  | MenC | 7.9; 3.4 – 18.2 | 4.2; 1.7 – 10.2 | 0.29 |
|  | MenW | 0.7; 0.3 – 1.7 | 0.3; 0.2 – 0.6 | 0.15 |
|  | MenY | 1.2; 0.5 – 2.8 | 0.8; 0.4 – 1.7 | 0.47 |
| **P* < 0.05  ^1^one missing observation | | | | |

**Supplementary Table 4.** **Geometric mean concentrations and 95% confidence intervals of meningococcal serogroup ACWY polysaccharide-specific serum IgG concentrations (µg/ml) in study participants reported for each disease type during follow-up.**

| Months | Serogroup | AAID | Immune deficiency | Uveitis | Sickle cell disease | *P* |
| --- | --- | --- | --- | --- | --- | --- |
| 0 |  | n = 10 | n = 11 | n = 5 | n = 0 |  |
|  | MenA | 0.1; 0.0 – 0.2 | 0.1;  0.0 – 0.6^1^ | 0.2; 0.0 – 1.4 | - | 0.61 |
|  | MenC | 0.4;  0.2 – 0.7 | 0.4;  0.1 – 1.5 | 1.2;  0.1 – 32.7 | - | 0.44 |
|  | MenW | 0.0;  0.0 – 0.0 | 0.1;  0.0 – 0.2 | 0.0;  0.0 – 0.1 | - | 0.19 |
|  | MenY | 0.0;  0.0 – 0.0 | 0.1;  0.0 – 0.6 | 0.1;  0.0 – 1.6 | - | 0.19 |
| 3-6 |  | n = 14 | n = 20 | n = 8 | n = 3 |  |
|  | MenA | 4.1;  1.2 – 13.4 | 7.3;  4.1 – 13.0 | 2.6;  0.7 – 9.9 | 15.8;  0.4 – 602.8 | 0.27 |
|  | MenC | 47.2;  17.7 – 125.6 | 36.2;  16.2 – 80.7 | 17.1;  4.5 – 65.8 | 194.3;  0.2 – 180592.8 | 0.23 |
|  | MenW | 1.6;  0.5 – 4.9 | 1.9;  0.8 – 4.4 | 0.5;  0.1 – 1.9 | 4.0;  0.3 – 59.1 | 0.20 |
|  | MenY | 2.8;  0.8 – 10.0 | 3.6;  1.7 – 7.5 | 1.5;  0.1 – 14.6 | 10.1;  3.2 – 32.0 | 0.52 |
| 12 |  | n = 16 | n = 17 | n = 10 | n = 4 |  |
|  | MenA | 2.1;  1.0 – 4.2 | 3.0;  1.4 – 6.1 | 1.6;  0.5 – 5.2 | 7.1;  1.0 – 49.7 | 0.31 |
|  | MenC | 20.9;  9.4 – 46.7 | 8.4;  4.1 – 17.3 | 12.1;  4.6 – 31.8^1^ | 53.3;  7.6 – 372.4 | 0.08 |
|  | MenW | 1.3;  0.5 – 3.7 | 1.3;  0.7 – 2.4 | 0.5;  0.1 – 2.1 | 2.5;  0.8 – 7.3 | 0.35 |
|  | MenY | 2.0;  0.7 – 6.0 | 2.6;  1.4 – 4.7 | 1.3;  0.2 – 9.0 | 4.0;  1.7 – 9.3 | 0.73 |
| 24 |  | n = 14 | n = 17 | n = 7 | n = 1 |  |
|  | MenA | 1.3;  0.5 – 3.2 | 1.3;  0.7 – 2.5 | 0.8;  0.2 – 3.1 | 7.0;  7.0 – 7.0 | 0.56 |
|  | MenC | 10.6;  4.4 – 25.5 | 2.4; 0.8 – 7.5 | 9.1;  3.3 – 25.2 | 7.4;  7.4 – 7.4 | 0.13 |
|  | MenW | 0.7;  0.3 – 1.9 | 0.4;  0.2 – 1.0 | 0.1;  0.0 – 0.5 | 1.0;  1.0 – 1.0 | 0.19 |
|  | MenY | 1.2;  0.4 – 3.1 | 1.0;  0.4 – 2.1 | 0.6;  0.1 – 4.4 | 2.5;  2.5 – 2.5 | 0.78 |
| Abbreviations: AAID = autoimmune and auto-inflammatory diseases  **P* < 0.05  ^1^one missing observation | | | | | | |
