# Supplementary material for: Meningococcal ACWY conjugate vaccine immunogenicity in adolescents with primary or secondary immune deficiencies, a prospective observational cohort study
Source: Pediatr Rheumatol Online J. 2023 Jul 20;21:73. doi: 10.1186/s12969-023-00846-3 (PMC10360259; doi:10.1186/s12969-023-00846-3)
Supplement: Supplementary file 2 — Supplementary Material 2 [file 12969_2023_846_MOESM2_ESM.docx]

Dear Editor,

Thank you for having our manuscript reviewed. Hereby we send you the responses to the reviewers comments.

Kind regards,

Marc Jansen

Reviewer reports

Reviewer #1: The study deals with immunogenicity of the Meningococcal ACWY conjugate vaccine in adolescents with secondary immune deficiencies, Although the patients are representing a very heterogenous group the results of the study are interesting and helpful.

*Comment: no comments to answer to.*

Reviewer #2: One of the interesting aspects of this study results from the practice of immunizing children with MenC conjugate vaccine in young children in the Netherlands, a practice not followed in every country (including the US). The study demonstrates that the study participants and the healthy controls have about the same level of antibody again the Men C PS as the healthy controls, and also achieve an equivalent boost in antibody against the MenC polysaccharide after MenACWY vaccine, while having less of a "primary" antibody response to the Men A and Men W Ps than the healthy controls (who also have higher baseline antibodies again the Men A and Men W PS). Does this suggest that a National Immunization Policy giving Men ACWY to infants could provide better protection to adolescents with primary and secondary immune deficiencies? Or is the better use of resources to focus on more frequent boosters in the patients with immune deficiencies, as the authors suggest?

As a follow up would the authors explain why the NIP in Netherlands is to give Men C conjugate vaccines to all young children?

*Comment: We thank the reviewer for the comments. We included the MenC vaccination in the national immunization program in the Netherlands in 2002 due to an outbreak of invasive meningococcal serogroup C disease (similar to, for example, the UK). In response to an increase in serogroup W disease in 2016-17, we now immunize all toddlers aged 14 months with MenACWY vaccination (replaced for the MenC vaccination) and included a dose at the age of 14 years as well in the NIP (*[Eurosurveillance | Increase of invasive meningococcal serogroup W disease in Europe, 2013 to 2017](https://www.eurosurveillance.org/content/10.2807/1560-7917.ES.2019.24.14.1800245;?crawler=true), [Vaccine Impact and Effectiveness of Meningococcal Serogroup ACWY Conjugate Vaccine Implementation in the Netherlands: A Nationwide Surveillance Study | Clinical Infectious Diseases | Oxford Academic](https://academic.oup.com/cid/article/74/12/2173/6370762)). *We indeed think that now we are giving MenACWY to infants, this could provide better protection to adolescents with primary and secondary immune deficiencies on the long run. However, in the next ten years, all adolescents receiving the MenACWY vaccination at the age of 14 will still be primed with only MenC. Therefore, we think that in the current situation, the focus should be to boosters in patients with immune deficiencies to reach optimal protection against the other subtypes, especially meningococcal W.*
